# Breaking down the Contradictory Roles of Histone Deacetylase SIRT1 in Human Breast Cancer

**DOI:** 10.3390/cancers10110409

**Published:** 2018-10-30

**Authors:** Khaldoun Rifaï, Mouhamed Idrissou, Frédérique Penault-Llorca, Yves-Jean Bignon, Dominique Bernard-Gallon

**Affiliations:** 1 Department of Oncogenetics, Centre Jean Perrin, CBRV, 28 place Henri-Dunant, 63001 Clermont-Ferrand, France; k.rifaii@live.com (K.R.); mouhamed.idrissou@etu.uca.fr (M.I.); Yves-Jean.BIGNON@clermont.unicancer.fr (Y.-J.B.); 2 INSERM—UMR 1240—Imagerie Moléculaire et Stratégies Théranostiques (IMoST), 58 Rue Montalembert, 63005 Clermont-Ferrand, France; Frederique.PENAULT-LLORCA@clermont.unicancer.fr; 3 Department of Biopathology, Centre Jean Perrin, 58 Rue Montalembert, 63011 Clermont-Ferrand, France

**Keywords:** breast cancer, SIRT1, deacetylation, epigenetic silencing, tumor promoter, tumor suppressor, SIRT1 modulators

## Abstract

Breast cancer (BC) is the most common type of cancer in women worldwide; it is a multifactorial genetic disease. Acetylation and deacetylation are major post-translational protein modifications that regulate gene expression and the activity of a myriad of oncoproteins. Aberrant deacetylase activity can promote or suppress tumorigenesis and cancer metastasis in different types of human cancers, including breast cancer. Sirtuin-1 (SIRT1) is a class-III histone deacetylase (HDAC) that deacetylates both histone and non-histone targets. The often-described ‘regulator of regulators’ is deeply implicated in apoptosis, gene regulation, genome maintenance, DNA repair, aging, and cancer development. However, despite the accumulated studies over the past decade, the role of SIRT1 in human breast cancer remains a subject of debate and controversy. The ambiguity surrounding the implications of SIRT1 in breast tumorigenesis stems from the discrepancy between studies, which have shown both tumor-suppressive and promoting functions of SIRT1. Furthermore, studies have shown that SIRT1 deficiency promotes or suppresses tumors in breast cancer, making it an attractive therapeutic target in cancer treatment. This review provides a comprehensive examination of the various implications of SIRT1 in breast cancer development and metastasis. We will also discuss the mechanisms underlying the conflicting roles of SIRT1, as well as its selective modulators, in breast carcinogenesis.

## 1. Introduction

Breast cancer is a genetically heterogeneous disease that remains the most commonly diagnosed malignancy amongst women worldwide. It is also the second leading cause of cancer death among females in developed countries after lung cancer [1]. Epigenetic alterations of proteins, histones, and chromatin play a fundamental role in gene expression regulation and ultimately, cancer formation. Reversible protein acetylation and deacetylation are amongst those alterations [2]. Histone deacetylases (HDACs) are major actors in gene expression regulation. By removal of acetyl groups from N-terminus tails of histones, HDACs repress the expression of genes implicated in the carcinogenesis process, such as oncogenes and tumor suppressor genes (TSGs). In addition to histones, HDACs regulate the expression and activity of a myriad of proteins involved in both cancer initiation and progression. Furthermore, aberrant expression of HDACs in various human cancers, and consequently their dysfunctional deacetylase activity, is deeply involved in the carcinogenesis process [3].

Sirtuins (SIRTs) are nicotinamide adenine dinucleotide (NAD+)-dependent class-III HDACs or lysine deacetylases (KDACs) that belong to the silent information regulator 2 (SIR2) family. The seven mammalian sirtuins (SIRT1–7) are key regulators in major biological processes, including cell death and survival, regulation of genomic stability, cellular senescence, metabolic regulation and inflammation [4,5]. Therefore, sirtuins have gained tremendous attention in the past decade in cancer research and numerous studies have demonstrated their direct implication in the carcinogenesis process of multiple human cancers [6]. Silent mating type information regulation 2 homolog 1 (Sirtuin-1) is the founding member of the sirtuin family and the most extensively studied. SIRT1 is expressed ubiquitously and is mainly found in the nucleus, but can shuttle between the nucleus and cytoplasm using its two nuclear localization signals and two nuclear export signals [7]. Due to its deacetylase activity, SIRT1 regulates a wide variety of fundamental cellular processes including apoptosis, DNA damage response and repair, cell differentiation and proliferation, chromatin remodeling and gene expression, endocrine signaling, aging, metabolism, stress response, and cancer development and metastasis [4,5,8,9,10]. Similar to most HDACs, aberrant SIRT1 expression is identified in numerous human malignancies and is directly linked to the tumorigenesis process and metastasis.

The implications of SIRT1 in breast cancer occurrence and development have been reported and largely studied over recent years, but its exact role in breast cancer remains very controversial and paradoxical so far. In fact, bifurcated SIRT1 can act as either a tumor suppressor or promoter in cancer cells. This highly context-specific role of SIRT1 in breast carcinoma seems to depend mainly on its upstream regulators or downstream substrates, as well as on its spatial distribution, cellular and molecular context, and tumor types. In this review, we summarize available data and give a general overview of the multiple implications of SIRT1 in breast tumorigenesis. We also explore the mechanisms underlying SIRT1 opposite functions in breast carcinogenesis. 

## 2. The Multifaceted Functions of HDAC SIRT1 in Cancer Biology

Other than histone deacetylation, the functional roles of SIRT1 are fulfilled by directly interacting with and deacetylating a wide range of downstream non-histone substrates, resulting in activation or repression of their catalytic activity. SIRT1 deacetylase activity regulates:

1. Tumor suppressors, including p53 [11], p73 [12], Forkhead transcription factors (FoXO) [13], E2F1 [14], and Rb (Retinoblastoma) [15].
2. Tumor promoters, including c-Myc [16], N-Myc [17], cortactin (CTTN) [18], NF-κB [19], β-catenin [20], and HIF-1α [21].
3. Chromatin-related enzymes, including p300 [22], hMOF and TIP60 [23], PCAF [24], HDAC1 [25], DNMT1 [26], SUV39H1 [27], and EZH2 (enhancer of Zest 2) [28].
4. Nuclear receptors and related factors, including estrogen receptor-alpha (ER-α) [29], androgen receptor (AR) [30], liver X receptor (LXR) [31], PPARγ [32], and PPARγ coactivator 1α (PGC-1α) [33].
5. DNA damage repair enzymes, such as Ku70 [34], XPC [35], XPA [36], APE1 [37], and WRN [38].

As a result of its diverse biological functions, multifaceted SIRT1 is critically implicated in the occurrence and progression of numerous human malignancies. Yet researchers have long been baffled by SIRT1 contradictory actions in the carcinogenesis process, and its involvement in cancer biology remains an open question.

## 3. SIRT1-Dependent Epigenetic Silencing via Histone Modification in Breast Carcinogenesis

SIRT1 lysine deacetylase activity regulates chromatin structure and transcription through epigenetic mechanisms [9]. Lysine acetylation of histones H3 and H4 is classically associated with transcriptional activation and increased gene expression. On the contrary, their deacetylation is generally associated with inactive chromatin and repression of gene expression [39]. In carcinogenesis, histone deacetylation leads to epigenetic silencing of various cancer-related genes; thus, HDACs could exert either tumor-promoting or tumor-suppressive roles depending on whether the repression happens in the genomic region of a tumor suppressor or a tumor promoter respectively. HDAC SIRT1 embodies these properties, and orchestrates the regulation of multiple cancer-related genes through histone deacetylation. Indeed, SIRT1 contributes to the epigenetic silencing by deacetylating H3 and H4 acetylated markers such as histones H3 lysine 4 (H3K4) [40], lysine 56 (H3K56) [41], lysine 9 (H3K9), lysine 14 (H3K14), and histone H4 lysine 16 (H4K16) [42,43]. In breast cancer, SIRT1-dependent epigenetic silencing of both oncogenes and TSGs is reported.

Pruitt et al. demonstrated that SIRT1 deficiency re-activates aberrantly silenced TSGs by increasing the acetylation of H3K9 and H4K16 epigenetic markers at their promoters in two breast cancer (BC) cell lines, indicating SIRT1-mediated epigenetic repression of TSGs through histone modifications in BC [44]. In contrast, Wang et al. revealed a tumor suppressor role of SIRT1 in BC. They reported that SIRT1 inhibits tumor growth in vivo by suppressing the expression of survivin, a member of the inhibitor of apoptosis (IAP) family that drives cell proliferation and viability [45]. SIRT1-mediated epigenetic silencing of survivin occurs through deacetylating the H3K9 marker on the survivin promoter, consequently suppressing its transcription in mammary tumors [46]. To address the confusion regarding SIRT1-dependent epigenetic regulation in BC pathogenesis, we characterized in recent studies an aspect of SIRT1 epigenetic behavior in human breast carcinoma. We showed that the opposite functions of SIRT1 in breast cancer are closely related to the molecular subtype. By modulating the acetylation status of key H3 and H4 epigenetic markers in a subtype-specific fashion, SIRT1 is more likely to exert an oncogenic role in luminal molecular subtypes and a tumor suppressor role in the triple-negative subtype (TNBC), also known as basal-like, both in vitro and ex vivo. Furthermore, we revealed that SIRT1 deficiency is associated with substantial induction of acetylated markers on six breast cancer-related gene promoters: *AR*, *BRCA1*, *ERS1*, *ERS2*, *EZH2*, and *EP300*, suggesting an active role of SIRT1 in regulating the expression of these genes in BC. We concluded that SIRT1 differential epigenetic regulation in breast cancer is predominantly governed by gene type and molecular subtype [40,47]. Other than BC, the duality of SIRT1 epigenetic regulation was also highlighted in colorectal cancer [48].

In fact, SIRT1-mediated epigenetic regulation extends to histone acetyltransferases (HATs), and other histone modifiers involved in transcription repression. SIRT1 fine-tuning of gene expression regulation is partly manifested through the repression of acetyltransferase activity of major HATs. Remarkably, these HATs acetylate the same histone targets as SIRT1. For instance, SIRT1 downregulates and blocks the activity of p300 of the p300/CBP family [22], hMOF and TIP60 of the MYST family [23], and PCAF of the GNAT family [24]. It stabilizes and stimulates the activity of HDACs, e.g., HDAC1 [25], DNA methyltransferases, e.g., DNMT1 deacetylated at Lys1349 and Lys1415 [26], and histone methyltransferases (HMTs), e.g., SUV39H1, deacetylated at Lys266 [27]. Furthermore, SIRT1 interacts and has a close functional relationship with EZH2, an essential HMT that constitutes the core catalytic subunit of polycomb repressive complex 2 (PRC2). SIRT1 and EZH2 form part of the PRC4 complex, along with other polycomb group proteins which are found overexpressed in breast cancer tumors. The two enzymes recruit SUV39H1 and DNMT1 to promote transcriptional repression at targeted genes [27,28,49,50]. Collectively, the data indicated that SIRT1 epigenetic regulation of gene expression is implemented by the means of regulation both, histone markers and their epigenetic ‘writers’ and ‘erasers’.

## 4. SIRT1 Assuming the Role of Tumor Promoter in Breast Carcinogenesis

The ‘guardian of the genome’ p53, a vital TSG that is frequently mutated in human tumors, was one of the first identified non-histone substrate of SIRT1 and the first evidence of SIRT1 implication in tumorigenesis. SIRT1 deacetylation of p53 at its Lys382 residue (p53K382) results in repression of p53-dependent apoptosis in response to DNA damage and promotes cell survival [11]. Upon DNA damage stimuli, SIRT1-mediated deacetylation of p53 is optimized by breast cancer metastasis suppressor 1 (BRMS1) [51]. BRMS1 potentiates SIRT1 activity through physically interacting with deleted in breast cancer 1 (DBC1), a negative regulator of SIRT1 [52]. SIRT1 also deacetylates and represses the activity of other damage-response enzymes, the mammalian forkhead transcription factors FoXO3a and FoXO4, inhibiting forkhead-dependent cell death [13,53]. In addition, SIRT1 binds to and inhibits the activity of E2F1, a tumor suppressor and apoptosis regulator, impairing its apoptotic functions [14]. SIRT1 also downregulates the activity of the tumor-suppressing retinoblastoma protein (Rb). Deacetylation of Rb by SIRT1 formed a domain similar to the SIRT1-targeted domain of p53, resulting in inhibiting Rb-dependent apoptosis [15]. Furthermore, SIRT1 overexpression in tumors is associated with upregulation of various oncoproteins. For example, SIRT1-dependent deacetylation of prototypic Myc oncogenes, c-Myc and N-Myc, enhances their stability and transcriptional activity, resulting in cancer cell survival and proliferation [16,17], respectively.

On the premise that SIRT1 is upregulated in various human cancers, SIRT1 could act as a tumor promoter. Since an abundance of SIRT1 expression is observed in breast tumors, many studies assert an oncogenic role of SIRT1 in breast carcinogenesis, and clinical studies demonstrated SIRT1 as a prognostic factor that significantly correlates with unfavorable clinicopathological factors. Actually, SIRT1 overexpression in breast tumors and mammary BC cell lines is significantly associated with lymph node metastasis, advanced TNM stage, low grade as per the modified Bloom–Richardson system, lymphovascular invasion, shorter disease-free survival (DFS), and overall survival (OS), luminal subtype, ER and PR expression, and is marginally associated with p53 loss [54,55,56]. Hence, SIRT1 upregulation is strongly correlated with breast tumorigenesis.

Xu et al. reported an upregulation of SIRT1 in breast tumors and (ER+) luminal BC cell line MCF-7. The authors revealed that SIRT1 upregulation promotes the proliferation, migration, and invasion of MCF-7 cells, whereas SIRT1 knockdown inhibits those effects. They showed that SIRT1 overexpression positively correlates with decreased expression of p53 and increased expression of DNA polymerase delta1 (*POLD1*) gene, an oncogene involved in genomic instability and cell proliferation; whilst the result of SIRT1 silencing is opposite. They concluded that SIRT1 is involved in breast carcinogenesis by inhibiting p53 and activating *POLD1* [57]. This was in line with a study by Jin et al. who revealed that SIRT1 upregulation significantly promotes breast cancer growth both in vitro and in vivo, whereas SIRT1 deficiency inhibits cancer cell proliferation. The authors showed that SIRT1 has effects on breast cancer cell growth through promoting the activity of oncogenic PI3K/Akt signaling pathway in vitro, and that SIRT1 is positively correlated with the expression of P-Akt in vivo [56]. SIRT1 is also involved in breast cancer progression and metastasis. Ota et al. demonstrated that SIRT1 inhibition by Sirtinol, a selective SIRT1 inhibitor, induces a senescence-like growth arrest in luminal cell line. The cellular senescence induced by SIRT1 inhibition co-occurs with impaired activation of oncogenic Ras–MAP kinase signaling pathways, implicated in cell growth and proliferation. These findings suggest an active role of SIRT1 in driving cell proliferation through Ras-MAP kinase signaling pathways [58]. Meanwhile, Zhang et al. found that SIRT1 and Cortactin; an oncogene associated with breast cancer metastasis), are more abundant in breast tumors than in their normal adjacent tissues. They showed that SIRT1-mediated deacetylation of cortactin promotes cell migration and breast tumorigenesis [18].

Meanwhile, SIRT1 oncogenic activity in BC is downregulated by different subclasses of miRNAs [59]. MiRNAs are small non-coding microRNAs that regulate the expression of many cancer-related genes. A recent study by Zou et al. reported that SIRT1 is negatively regulated by miR-22, a subclass of miRNAs, in the ER+ MCF-7 cell line. The authors showed that an ectopic expression of miR-22 reduces the proliferation, migration and invasion of MCF-7 cells, whereas SIRT1 overexpression eliminates the suppressive effects of miR-22. They concluded that miR-22 inhibitory effects are partly fulfilled by downregulating SIRT1 expression in vitro [60]. A similar study by Zhang et al. confirmed SIRT1 as a direct target of miR-22 in both (ER+) and (ER−) cell lines. The authors showed that SIRT1 knockdown induces apoptosis, inhibits tumorigenesis, and enhances radiosensitivity of breast cancer cells. In addition, miR-22 overexpression suppresses tumorigenesis and improves radiosensitivity of breast cancer cells by targeting SIRT1 in vitro. They concluded on the same note as Zou et al. [61]. 

SIRT1 is a confirmed target of another subclass of miRNAs. MiR-34a represses SIRT1 expression through a miR-34a-binding site within the 3′UTR of SIRT1. MiR-34a-mediated inhibition of SIRT1 leads to an increase of acetylated p53 and consequently, increased expression of pro-apoptotic genes *p21* and *PUMA* in colon cancer cells [62]. In breast cancer, an ectopic expression of miR-34a inhibits the growth of breast cancer cells by inducing apoptosis and suppressing cell migration in both ER+ and ER− cell lines. It was revealed that miR-34a tumor-suppressive role is partly implemented by the means of suppressing SIRT1 expression in vitro [63]. Another study showed that SIRT1 downregulation or miR-34a upregulation inhibits cell proliferation and colony formation ability in the MCF-7 cell line, as well as in CD44+/CD24− breast cancer stem cells (BCSCs). SIRT1 knockdown in BCSCs positively correlates with decreased expression of BCSCs markers: ALDH1, BMI1, and NANOG. In addition, a stable expression of miR-34a or silencing of SIRT1 reduces tumor growth in nude mice xenografts. SIRT1 downregulation also positively correlates with decreased ALDH1 in vivo. It is postulated then, that miR-34a upregulation suppresses the proliferative potential of BCSCs in vitro and in vivo by partially downregulating SIRT1 [64]. The diverse tumor-promoting properties of SIRT1 in breast cancer are resumed in Table 1.

## 5. SIRT1 Assuming the Role of Tumor Suppressors in Breast Carcinogenesis

Alternatively, there is much convincing evidence supporting a tumor suppressive role of SIRT1 in carcinogenesis, considering its implication in maintaining genome integrity via chromatin regulation and DNA damage response. Following DNA damage, SIRT1 regulates and optimizes DNA repair pathways, and is required for efficient single-strand and double-strand DNA breaks (DSB) repair [65]. SIRT1 stabilizes and upregulates the activity of DNA damage repair enzymes including Ku70 [34], XPC [35], XPA [36], APE1 [37] and WRN [38]. Aside from regulating genome stability, SIRT1 represses the expression of oncogenes through epigenetic silencing, and downregulates the activity of oncoproteins through direct deacetylation. For example, SIRT1 downregulates the transcriptional activity of the NF-kappaB-dependent cell survival pathway through physically interacting and deacetylating the RelA/p65 subunit of NF-kappaB at lysine 310 (NF-κB K310) [19]. SIRT1 also impairs the oncogenic activity of the Wnt/β-catenin signaling pathway. Aberrant activation of this pathway in various cancers promotes the transcription of many oncogenes through the transcriptional activity of β-catenin. SIRT1-mediated deacetylation of β-catenin suppresses its ability to activate transcription and drive cell proliferation [20]. 

In breast cancer, Wang et al. asserted a tumor suppressor role of SIRT1 through its implication in DNA damage response and genome integrity. The authors revealed that SIRT1 haploinsufficiency in SIRT1^+/−^ p53^+/−^ mice facilitates tumorigenesis, whereas SIRT1 activation by resveratrol, a bona fide activator of SIRT1 [66], reduces tumorigenesis in vivo. Moreover, by mutating the *SIRT1* gene, they found that SIRT1-null mice embryos die during embryonic development and that SIRT1 deficiency causes genetic instability and impaired DNA damage repair. The authors also found an increased expression of anti-apoptotic oncoproteins Bcl-2 and survivin in SIRT1-null embryos [67]. To investigate this observation, Wang et al. conducted another study on human BRCA1-associated breast cancers. The authors noticed that lack of BRCA1 in BRCA1-mutant breast tumors is associated with reduced expression of SIRT1 and high levels of survivin, and showed BRCA1 to positively regulate SIRT1 expression in vitro. They also demonstrated that SIRT1 activation by Resveratrol blocks cell proliferation and antagonizes tumor growth through downregulating survivin expression in vivo [46]. Paradoxically, survivin is also repressed by wild-type p53 [45], the latter being a certified target of SIRT1 [11]. 

The interplay between SIRT1 and BRCA1 in BC is uncovered in another study. Zhang et al. revealed that BRCA1 induction suppresses AR-dependent tumor growth through SIRT1 activation in both (ER^+^) and (ER^−^) cell lines. They showed that resveratrol inhibits AR–stimulated proliferation by activating SIRT1 in vitro, and that SIRT1 overexpression in xenograft model BALB/c mice represses tumor growth in vivo. They concluded on the note that SIRT1 inhibits breast cancer development through diverse cellular processes [68], further establishing SIRT1 tumor-suppressive properties in breast cancer. In fact, the direct functional link of SIRT1 with AR was previously characterized by Fu et al. who revealed that SIRT1 binds to and downregulates AR activity in vitro. They showed that SIRT1-mediated repression of AR activity inhibits androgen-induced cell proliferation in prostate cancer [30]. A recent study by Yu et al. showed that an ectopic expression of SIRT1 in mesenchymal stem cells (MSCs) effectively suppresses breast tumor growth by inhibiting proliferation and inducing apoptosis in vivo. The authors found that SIRT1-induced antitumor activity in MSCs is achieved by increasing CXCL10 expression, a chemotactic factor necessary for the recruitment of the antitumor natural killer (NK) cells. They showed that breast tumor suppression is carried out through the actions of CXCL10-recruited NK cells [69]. 

In addition, SIRT1 reduces drug-resistance in breast cancer. A well-structured study by Shi et al. reported that SIRT1 deficiency induces chemo-resistance to paclitaxel (PTX), a chemotherapy drug used to treat BC, by disrupting the SIRT1-PRRX1-KLF4 axis which regulates chemo-resistance. The authors found that SIRT1 depletion destabilizes PRRX1 and leads to KLF4 upregulation, a core stemness factor that promotes carcinogenesis. KLF4 subsequently promotes transcription of ALDH1, which induces BCSCs, confers cellular resistance to chemotherapy, and promotes distant metastasis [70]. SIRT1 was also shown to reduce drug-resistance to tamoxifen (TAM), a widely used drug in the treatment of luminal BC. Li et al. revealed that SIRT1 silencing leads to TAM-resistance in luminal MCF-7 cell line (TAMR-MCF-7 cells), whereas SIRT1 restoration compromised brachyury-mediated TAM-resistance. The authors demonstrated that the overexpression of brachyury, a molecular mediator of resistance to tamoxifen, enhances TAM-resistance by increasing cell viability, reducing cell apoptosis, and downregulating SIRT1 expression in vitro. They concluded that brachyury mediates TAM-resistance by downregulating SIRT1 expression [71]. However, a study by Choi et al. postulated that SIRT1 overexpression contributes to TAM-resistance in MCF-7 cells by activating FoxO1 (Forkhead box-containing protein, O subfamily1), which in turn upregulates the expression of MRP2 (multidrug resistance protein 2) in TAMR-MCF-7 cells [72]. The diverse tumor-suppressive properties of SIRT1 in breast cancer are resumed in Table 2.

## 6. The Functional Duality of SIRT1 in Breast Cancer

Conflicting studies concerning SIRT1 ambiguous involvement in breast cancer extend to many aspects of the disease.

### 6.1. SIRT1 Role in ER-α-Positive Luminal BC Molecular Subtypes

**Figure 1:**
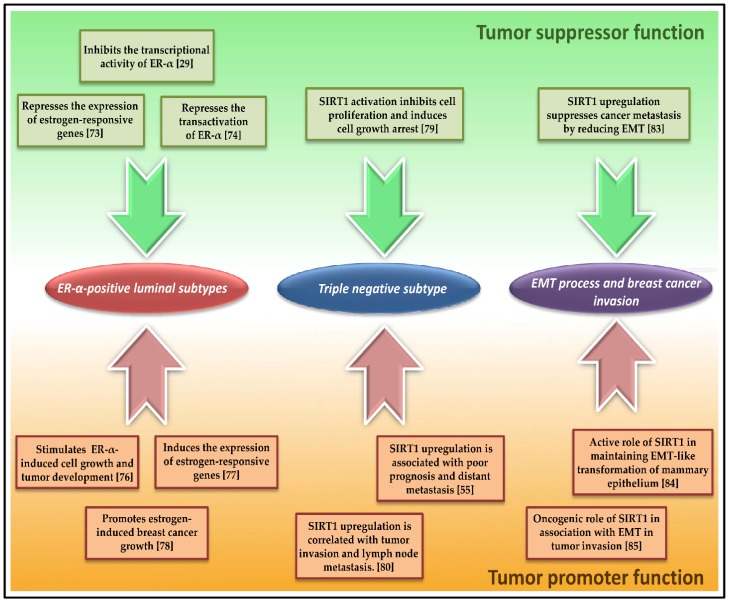
Bifurcated functions of SIRT1 in breast carcinogenesis. ER: estrogen receptor; EMT: epithelial-to-mesenchymal transition.

The oncogenic estrogen/ER-α-mediated signaling pathways stimulate cell proliferation and tumor growth in luminal hormone-dependent subtypes, through the activation of estrogen-responsive genes by ER-α transcriptional activity. Yu et al. revealed that SIRT1 binds to and inhibits the transcriptional activity of ER-α by regulating its acetylation status. They showed that SIRT1 represses the co-activator synergy between DBC1 and CCAR1, ER-α co-activators that enhance its transcriptional activity. They asserted SIRT1 as a major regulator of ER-α activity and co-activator synergy [29]. Meanwhile, Moore et al. reported that SIRT1 inhibits tumor cell reaction to estrogen in vitro. The authors showed that SIRT1 represses basal and inducible expression of estrogen-responsive genes, while inhibition of SIRT1 activity results in transcriptional activation of estrogen-responsive genes and consequently, cancer cell proliferation. They demonstrated that SIRT1-mediated repression of the proliferative response to estrogens is ER-α-dependent. They concluded that SIRT1 downregulates the ER-mediated signaling pathway in BC cells [73]. A more recent study by Xu et al. showed that SIRT1-mediated deacetylation of ER-α represses the transactivation of ER-α and consequently, inhibits the proliferation of BC cells in vitro. The authors showed that checkpoint suppressor 1 (CHES1) interacts with ER-α and enhances the recruitment of SIRT1, thus enabling SIRT1-mediated repression of ER-α transactivation and impairing ER-α transcriptional activity [74]. Furthermore, the SIRT1 activator resveratrol has been reported to suppress estrogen-dependent growth of luminal BC cells [75]. These studies demonstrated an anti-tumor role of SIRT1 in luminal subtypes through impairing ER-mediated signaling pathways (Figure 1).

On the other hand, alternative studies reported an oncogenic role of SIRT1 in luminal breast tumors. Elangovan et al. revealed that SIRT1 is activated and upregulated by ER-α in response to estrogens. They showed that ER-α physically binds to and functionally cooperates with SIRT1 toward the stimulation of breast tumor cells. In addition, SIRT1 inactivation eliminates estrogen/ER-α-induced cell growth and tumor development, triggering apoptosis and cell growth arrest. The authors concluded that SIRT1 is required for estrogen-induced breast cancer growth [76]. Another study by Yao et al. demonstrated that SIRT1 deficiency suppresses ER-α expression and leads to inhibition of estrogen-responsive gene expression in vitro. They showed that SIRT1 deficiency downregulates ER-α-mediated estrogen response genes in vivo, impairing ER-α-mediated signaling pathways in breast tumors. They postulated that SIRT1 may be a co-activator of ER-α in breast cancer [77]. In accordance with these findings, Santolla et al. investigated the expression and function of SIRT1 by estrogens in ER-negative BC cells and cancer-associated fibroblasts (CAFs). The authors showed that estrogens upregulate SIRT1 expression through GPER (G protein-coupled ER) along with subsequent activation of the oncogenic EGFR/ERK/c-fos/AP-1 transduction pathway in vitro. They demonstrated that SIRT1 and GPER promote tumor growth both in vitro and in vivo. The authors then asserted a pro-survival role of SIRT1 and its implication in the prevention of apoptosis and cell cycle arrest [78].

### 6.2. SIRT1 Role in Non-Hormone-Dependant Triple-Negative Subtype (TNBC)

There are also contrasting studies concerning SIRT1 biological role in the TNBC subtype. Yi et al. reported that SIRT1 activation by a SIRT1 specific activator YK-3-237, induces the deacetylation of mt-p53, the oncogenic mutant form of p53, Deacetylation of mt-p53 upregulates the expression of wild-type p53-targets the *PUMA* and *NOXA* pro-apoptotic genes, suppressing cell proliferation and arresting cell growth of TNBC cell lines [79]. On the other hand, Wu et al. asserted an oncogenic role of SIRT1 in TNBC subtype. They revealed that an increased expression of SIRT1 is associated with poor prognosis, shorter DFS and OS, and distant metastasis in both TNBC and non-TNBC subtypes [55]. These findings are in agreement with those of Chung et al. who reported that SIRT1 upregulation positively correlates with tumor invasion and lymph node metastasis. They also showed that *SIRT1* gene silencing with SIRT1-siRNA significantly reduces the invasion ability of transfected versus non-transfected TNBC cell lines. The authors suggested the potential role of SIRT1 as a prognostic indicator, as well as a novel therapeutic target in triple negative BC [80]. Interestingly, a recent study by Urra et al. showed that SIRT1-mediated activation of AMPK selectively inhibits fibronectin-dependent migration of TNBC cells. However, the activation of SIRT1/AMPK axis has a cyto-protective effect in TNBC cells, promoting cell survival and proliferation but suppressing their ability to migrate. The authors demonstrated that SIRT1/AMPK activation impairs cell migration by reducing β1-integrin, a key protein involved in fibronectin-stimulated cell migration, on the cell surface and in turn, reduces cellular adhesion to the extracellular matrix [81].

### 6.3. SIRT1 Implication in the Epithelial-to-Mesenchymal Transition (EMT) Process, and Breast Cancer Invasion and Metastasis

The EMT process refers to the transformation of an epithelial cell to a mesenchymal cell; the process results in repressed E-cadherin expression and loss of cell-adhesive properties of epithelial cells. It also prevents apoptosis, and is critically implicated in cancer invasion and metastasis [82]. Using a xenograft mouse model, Simic et al. analyzed the metastatic potential of BC cells with or without SIRT1 in vivo. They found that SIRT1 upregulation suppresses cancer metastasis by reducing EMT, consequently maintaining E-cadherin expression; whereas SIRT1 repression promotes metastasis of breast epithelial cells in an orthotopic model of breast cancer. The authors also demonstrated that SIRT1 restrains the transforming-growth-factor (TGF)-β-signaling pathway that drives EMT. They postulated that SIRT1 suppression leads to E-cadherin degradation from the cell surface, thereby releasing β-catenin from the cadherin junctions to the nucleus, which is the characteristic of mesenchymal cells [83], thus asserting SIRT1 tumor-suppressive properties in the EMT process of BC. In contrast, Eades et al. reported that SIRT1 is overexpressed upon EMT-like transformation of human mammary cells in vitro, and that TGF-β-induced EMT leads to SIRT1 overexpression in epithelial cells. They also observed an increased SIRT1 recruitment to the E-cadherin promoter, resulting in SIRT1-mediated epigenetic silencing of E-cadherin, while SIRT1 knockdown restores E-cadherin expression. The authors also showed that SIRT1 deficiency prevents transformation of mammary epithelial cells by decreasing anchorage-independent growth and cell migration in vitro, hence indicating SIRT1 role in maintaining EMT-like transformation of the mammary epithelium [84]. Another study by Jin et al. revealed that SIRT1 expression is significantly correlated with increased expression of EMT-related proteins, vimentin and snail-1, and reduced expression of E-cadherin in triple-negative breast tumors; whereas inhibition of SIRT1 has opposite effects in vitro. They showed that SIRT1 inhibition also reduces the invasion ability of TNBC cell lines in vitro. The authors then suggested an oncogenic role of SIRT1 in association with EMT in tumor invasion of TNBC subtype [85].

## 7. SIRT1 Modulators towards Breast Cancer Treatment

Being a key regulator of numerous cancer-associated processes, SIRT1 has been the subject of intense research in recent years. As a consequence, countless studies investigated/reviewed the therapeutic potential of SIRT1 in cancer treatment, and a plethora of small chemical compounds that modulate SIRT1 activity were discovered and patented [86,87,88,89]. These modulators (i.e., activators/inhibitors) not only enabled researchers to have a greater understanding of SIRT1 biological function and regulatory mechanisms, but also showed promising therapeutic applications in clinical trials for various human diseases, such as metabolic disorders, cardiovascular and neurodegenerative diseases, endothelial dysfunctions, inflammation, and cancer [90,91,92]. Although SIRT1 modulators have proven their efficiency in cancer cells by reducing cell viability and inducing apoptosis, their therapeutic functions remain utterly related to the role and expression rate of SIRT1 in a specific cancer, which in turn may vary drastically as we previously described. 

While SIRT1 activators were initially favored as calorie restriction mimetics, researchers demonstrated their beneficial effects in delaying age-related decline in heart function and neuronal loss, also in preventing tumorigenesis. Resveratrol, a polyphenol described as an anti-aging drug and calorie restriction mimetic, was amongst the first characterized activators of SIRT1 [66,93]. In breast cancer, we previously showed that SIRT1 activation by resveratrol in SIRT1^+/−^ p53^+/−^ mice reduces tumorigenesis in vivo [67], as well as AR–stimulated proliferation [68]. Also, resveratrol was shown to repress estrogen-dependent growth by impairing ER-α-mediated signaling pathways [75]. Due to shortage in resveratrol bioavailability, synthetic compounds that are structurally unrelated to resveratrol but 1000-fold more potent were synthesized and collectively named SIRT1-activating compounds (STAC) [94]. These STACs are currently being used as SIRT1 activators in breast cancer studies; they include among others SRT1460, SRT1720, SRT2104, and SRT2183 [86,87,91].

SIRT1 inhibitors have shown their therapeutic potentials in the treatment of various pathologies such as immunodeficiency virus infections, parasitic diseases, Parkinson’s disease, and cancer therapy. Since SIRT1 is upregulated in multiple types of cancer, anticancer studies were more focused on SIRT1 inhibitors compared to SIRT1 activators [87,92]. As a result, a wide range of pharmacological inhibitory molecules were designed and tested such as sirtinol, salermide, splitomicin, cambinol, suramin, tenovin, nicotinamide, indole derivatives, and their structurally similar analogs. In breast cancer, in vitro and in vivo studies on ER^+^ and ER^−^ cell lines showed that SIRT1 inhibition by these molecules suppresses cancer cell proliferation and induces p53-mediated apoptosis through increasing the acetylation of its Lys382 (p53K382), or in some cases, induces p53-independent apoptosis by reactivating proapoptotic genes (such as *CASP* genes that encode for caspase-3/8/9) that were epigenetically repressed by SIRT1 [95,96,97,98,99,100], thus proving the antitumor activity of SIRT1 inhibitors in BC.

## 8. Conclusions and Future Directions

In conclusion, regardless of whether SIRT1 has a pro-survival role by repressing TSGs, upregulating the expression of oncogenes, and activating oncogeneic signaling pathways such as PI3K/Akt and Ras-MAP kinase, or whether it has a proapoptotic role by reducing tumorigenesis and AR-mediated proliferation, downregulating the expression of oncogenes, and participating in ER-α-mediated signaling pathways and the EMT process, there is no doubt as to its significant role in breast carcinogenesis. Studies showed that SIRT1 plays different roles according to different BC molecular subtypes. Since BC is characterized by its molecular and clinical heterogeneity, with variations in gene expression profiles compared to intrinsic subtypes, one might argue that researchers should take into account the molecular classification of used human mammary tumors and cell lines in their future studies. Further investigations should also include a statistically sufficient sample size, and use of multiple cell lines in the same study. Nonetheless, considerable progress has been made in this research area in the last 10 years. SIRT1 modulators have been discovered or designed, and clinical studies investigating the therapeutic potential of SIRT1 in cancer treatment hold promising results. Thus, this research field should be prioritized and more large-scale studies are needed in order to decipher the code of the enzymatic duality of SIRT1 in breast carcinogenesis.

## Figures and Tables

**Table 1:** Mechanisms of action of SIRT1 tumor-promoting functions in breast carcinogenesis. SIRT1: sirtuin-1; TSG: tumor suppressor gene; POLD1: DNA polymerase delta1; BC: breast cancer; BCSC: breast cancer stem cell.

Mechanism of Action	References
SIRT1 represses TSG expression through epigenetic silencing	[44]
SIRT1 upregulation positively correlates with p53 downregulation and POLD1 upregulation	[57]
SIRT1 stimulates the activation of PI3K/Akt signaling pathway	[56]
SIRT1 downregulation co-occurred with impaired activation of Ras-MAPK signaling pathway	[58]
SIRT1-mediated deacetylation of cortactin promotes cell migration	[18]
SIRT1 upregulation eliminates the tumor-suppressive effects of miR-22	[60]
SIRT1 downregulation induces apoptosis and enhances radiosensitivity of BC cells	[61]
SIRT1 downregulation by miR-34a suppresses proliferation and migration of BC cells	[63]
SIRT1 downregulation in BCSCs positively correlates with decreased expression of BCSCs markers and reduces tumor growth in nude mice xenografts	[64]

**Table 2:** Mechanisms of action of SIRT1 tumor-suppressive functions in breast carcinogenesis. AR: androgen receptor; MSC: mesenchymal stem cell; NK: natural killer.

Mechanism of Action	References
SIRT1 upregulation by resveratrol reduces breast tumorigenesis in vivo SIRT1 loss causes genetic instability and impaired DNA damage repair SIRT1 loss positively correlates with an increased expression of oncoproteins Bcl-2 and survivin	[67]
SIRT1 upregulation antagonizes tumor growth by downregulating survivin expression in vivo SIRT1 represses survivin expression through epigenetic silencing	[46]
SIRT1 upregulation inhibits AR–stimulated proliferation in vitro SIRT1 upregulation represses tumor growth in xenograft BALB/c mice	[68]
SIRT1 upregulation in MSCs suppresses tumor growth in vivo through CXCL10-recruited NK cells	[69]
SIRT1 downregulation causes chemo-resistance by impairing SIRT1-PRRX1-KLF4 axis	[70]
SIRT1 downregulation induces brachyury-mediated tamoxifen-resistance in the luminal cell line	[71]

## References

[1] Torre L.A., Bray F., Siegel R.L., Ferlay J., Lortet-Tieulent J., Jemal A. (2015). Global cancer statistics, 2012. CA Cancer J. Clin..

[2] Esteller M. (2008). Epigenetics in cancer. N. Engl. J. Med..

[3] Ropero S., Esteller M. (2007). The role of histone deacetylases (HDACs) in human cancer. Mol. Oncol..

[4] Haigis M.C., Sinclair D.A. (2010). Mammalian sirtuins: Biological insights and disease relevance. Annu. Rev. Pathol..

[5] Saunders L.R., Verdin E. (2007). Sirtuins: Critical regulators at the crossroads between cancer and aging. Oncogene.

[6] Martinez-Pastor B., Mostoslavsky R. (2012). Sirtuins, Metabolism, and Cancer. Front. Pharmacol..

[7] Tanno M., Sakamoto J., Miura T., Shimamoto K., Horio Y. (2007). Nucleocytoplasmic Shuttling of the NAD^+^-Dependent Histone Deacetylase SIRT1. J. Biol. Chem..

[8] Houtkooper R.H., Pirinen E., Auwerx J. (2012). Sirtuins as regulators of metabolism and healthspan. Nat. Rev. Mol. Cell Biol..

[9] Bosch-Presegué L., Vaquero A. (2015). Sirtuin-dependent epigenetic regulation in the maintenance of genome integrity. FEBS J..

[10] Liu T., Liu P.Y., Marshall G.M. (2009). The critical role of the class III histone deacetylase SIRT1 in cancer. Cancer Res..

[11] Vaziri H., Dessain S.K., Ng Eaton E., Imai S.I., Frye R.A., Pandita T.K., Guarente L., Weinberg R.A. (2001). hSIR2(SIRT1) functions as an NAD-dependent p53 deacetylase. Cell.

[12] Dai J.M., Wang Z.Y., Sun D.C., Lin R.X., Wang S.Q. (2007). SIRT1 interacts with p73 and suppresses p73-dependent transcriptional activity. J. Cell. Physiol..

[13] Motta M.C., Divecha N., Lemieux M., Kamel C., Chen D., Gu W., Bultsma Y., McBurney M., Guarente L. (2004). Mammalian SIRT1 represses forkhead transcription factors. Cell.

[14] Wang C., Chen L., Hou X., Li Z., Kabra N., Ma Y., Nemoto S., Finkel T., Gu W., Cress W.D. (2006). Interactions between E2F1 and SIRT1 regulate apoptotic response to DNA damage. Nat. Cell Biol..

[15] Wong S., Weber J.D. (2007). Deacetylation of the retinoblastoma tumour suppressor protein by SIRT1. Biochem. J..

[16] Menssen A., Hydbring P., Kapelle K., Vervoorts J., Diebold J., Lüscher B., Larsson L.-G., Hermeking H. (2012). The c-MYC oncoprotein, the NAMPT enzyme, the SIRT1-inhibitor DBC1, and the SIRT1 deacetylase form a positive feedback loop. Proc. Natl. Acad. Sci. USA.

[17] Marshall G.M., Liu P.Y., Gherardi S., Scarlett C.J., Bedalov A., Xu N., Iraci N., Valli E., Ling D., Thomas W. (2011). SIRT1 Promotes N-Myc Oncogenesis through a Positive Feedback Loop Involving the Effects of MKP3 and ERK on N-Myc Protein Stability. PLoS Genet..

[18] Zhang Y., Zhang M., Dong H., Yong S., Li X., Olashaw N., Kruk P.A., Cheng J.Q., Bai W., Chen J. (2009). Deacetylation of cortactin by SIRT1 promotes cell migration. Oncogene.

[19] Yeung F., Hoberg J.E., Ramsey C.S., Keller M.D., Jones D.R., Frye R.A., Mayo M.W. (2004). Modulation of NF-kappaB-dependent transcription and cell survival by the SIRT1 deacetylase. EMBO J..

[20] Firestein R., Blander G., Michan S., Oberdoerffer P., Ogino S., Campbell J., Bhimavarapu A., Luikenhuis S., de Cabo R., Fuchs C. (2008). The SIRT1 deacetylase suppresses intestinal tumorigenesis and colon cancer growth. PLoS ONE.

[21] Lim J.-H., Lee Y.-M., Chun Y.-S., Chen J., Kim J.-E., Park J.-W. (2010). Sirtuin 1 modulates cellular responses to hypoxia by deacetylating hypoxia-inducible factor 1α. Mol. Cell.

[22] Bouras T., Fu M., Sauve A.A., Wang F., Quong A.A., Perkins N.D., Hay R.T., Gu W., Pestell R.G. (2005). SIRT1 deacetylation and repression of p300 involves lysine residues 1020/1024 within the cell cycle regulatory domain 1. J. Biol. Chem..

[23] Peng L., Ling H., Yuan Z., Fang B., Bloom G., Fukasawa K., Koomen J., Chen J., Lane W.S., Seto E. (2012). SIRT1 negatively regulates the activities, functions, and protein levels of hMOF and TIP60. Mol. Cell. Biol..

[24] Fulco M., Schiltz R.L., Iezzi S., King M.T., Zhao P., Kashiwaya Y., Hoffman E., Veech R.L., Sartorelli V. (2003). Sir2 regulates skeletal muscle differentiation as a potential sensor of the redox state. Mol. Cell.

[25] Dobbin M.M., Madabhushi R., Pan L., Chen Y., Kim D., Gao J., Ahanonu B., Pao P.-C., Qiu Y., Zhao Y. (2013). SIRT1 collaborates with ATM and HDAC1 to maintain genomic stability in neurons. Nat. Neurosci..

[26] Peng L., Yuan Z., Ling H., Fukasawa K., Robertson K., Olashaw N., Koomen J., Chen J., Lane W.S., Seto E. (2011). SIRT1 deacetylates the DNA methyltransferase 1 (DNMT1) protein and alters its activities. Mol. Cell. Biol..

[27] Vaquero A., Scher M., Erdjument-Bromage H., Tempst P., Serrano L., Reinberg D. (2007). SIRT1 regulates the histone methyl-transferase SUV39H1 during heterochromatin formation. Nature.

[28] Wan J., Zhan J., Li S., Ma J., Xu W., Liu C., Xue X., Xie Y., Fang W., Chin Y.E. (2015). PCAF-primed EZH2 acetylation regulates its stability and promotes lung adenocarcinoma progression. Nucleic Acids Res..

[29] Yu E.J., Kim S.-H., Heo K., Ou C.-Y., Stallcup M.R., Kim J.H. (2011). Reciprocal roles of DBC1 and SIRT1 in regulating estrogen receptor α activity and co-activator synergy. Nucleic Acids Res..

[30] Fu M., Liu M., Sauve A.A., Jiao X., Zhang X., Wu X., Powell M.J., Yang T., Gu W., Avantaggiati M.L. (2006). Hormonal control of androgen receptor function through SIRT1. Mol. Cell. Biol..

[31] Li X., Zhang S., Blander G., Tse J.G., Krieger M., Guarente L. (2007). SIRT1 deacetylates and positively regulates the nuclear receptor LXR. Mol. Cell.

[32] Han L., Zhou R., Niu J., McNutt M.A., Wang P., Tong T. (2010). SIRT1 is regulated by a PPAR{γ}-SIRT1 negative feedback loop associated with senescence. Nucleic Acids Res..

[33] Rodgers J.T., Lerin C., Haas W., Gygi S.P., Spiegelman B.M., Puigserver P. (2005). Nutrient control of glucose homeostasis through a complex of PGC-1alpha and SIRT1. Nature.

[34] Jeong J., Juhn K., Lee H., Kim S.-H., Min B.-H., Lee K.-M., Cho M.-H., Park G.-H., Lee K.-H. (2007). SIRT1 promotes DNA repair activity and deacetylation of Ku70. Exp. Mol. Med..

[35] Ming M., Shea C.R., Guo X., Li X., Soltani K., Han W., He Y.-Y. (2010). Regulation of global genome nucleotide excision repair by SIRT1 through xeroderma pigmentosum C. Proc. Natl. Acad. Sci. USA.

[36] Fan W., Luo J. (2010). SIRT1 regulates UV-induced DNA repair through deacetylating XPA. Mol. Cell.

[37] Yamamori T., DeRicco J., Naqvi A., Hoffman T.A., Mattagajasingh I., Kasuno K., Jung S.-B., Kim C.-S., Irani K. (2010). SIRT1 deacetylates APE1 and regulates cellular base excision repair. Nucleic Acids Res..

[38] Uhl M., Csernok A., Aydin S., Kreienberg R., Wiesmüller L., Gatz S.A. (2010). Role of SIRT1 in homologous recombination. DNA Repair (Amst.).

[39] Kouzarides T. (2007). Chromatin modifications and their function. Cell.

[40] Rifaï K., Judes G., Idrissou M., Daures M., Bignon Y.-J., Penault-Llorca F., Bernard-Gallon D. (2018). SIRT1-dependent epigenetic regulation of H3 and H4 histone acetylation in human breast cancer. Oncotarget.

[41] Das C., Lucia M.S., Hansen K.C., Tyler J.K. (2009). CBP/p300-mediated acetylation of histone H3 on lysine 56. Nature.

[42] Vaquero A., Scher M., Lee D., Erdjument-Bromage H., Tempst P., Reinberg D. (2004). Human SirT1 interacts with histone H1 and promotes formation of facultative heterochromatin. Mol. Cell.

[43] Imai S., Armstrong C.M., Kaeberlein M., Guarente L. (2000). Transcriptional silencing and longevity protein Sir2 is an NAD-dependent histone deacetylase. Nature.

[44] Pruitt K., Zinn R.L., Ohm J.E., McGarvey K.M., Kang S.-H.L., Watkins D.N., Herman J.G., Baylin S.B. (2006). Inhibition of SIRT1 Reactivates Silenced Cancer Genes without Loss of Promoter DNA Hypermethylation. PLoS Genet..

[45] Altieri D.C. (2008). Survivin, cancer networks and pathway-directed drug discovery. Nat. Rev. Cancer.

[46] Wang R.-H., Zheng Y., Kim H.-S., Xu X., Cao L., Luhasen T., Lee M.-H., Xiao C., Vassilopoulos A., Chen W. (2008). Interplay among BRCA1, SIRT1, and Survivin during BRCA1-associated tumorigenesis. Mol. Cell.

[47] Rifaï K., Judes G., Idrissou M., Daures M., Bignon Y.-J., Penault-Llorca F., Bernard-Gallon D. (2017). Dual SIRT1 expression patterns strongly suggests its bivalent role in human breast cancer. Oncotarget.

[48] Rifaï K., Idrissou M., Daures M., Bignon Y.-J., Penault-Llorca F., Bernard-Gallon D. (2018). SIRT1 in Colorectal Cancer: A Friend or Foe?. OMICS A J. Integr. Biol..

[49] Kuzmichev A., Margueron R., Vaquero A., Preissner T.S., Scher M., Kirmizis A., Ouyang X., Brockdorff N., Abate-Shen C., Farnham P. (2005). Composition and histone substrates of polycomb repressive group complexes change during cellular differentiation. Proc. Natl. Acad. Sci. USA.

[50] Viré E., Brenner C., Deplus R., Blanchon L., Fraga M., Didelot C., Morey L., Van Eynde A., Bernard D., Vanderwinden J.-M. (2006). The Polycomb group protein EZH2 directly controls DNA methylation. Nature.

[51] Liu X., Ehmed E., Li B., Dou J., Qiao X., Jiang W., Yang X., Qiao S., Wu Y. (2016). Breast cancer metastasis suppressor 1 modulates SIRT1-dependent p53 deacetylation through interacting with DBC1. Am. J. Cancer Res..

[52] Zhao W., Kruse J.-P., Tang Y., Jung S.Y., Qin J., Gu W. (2008). Negative regulation of the deacetylase SIRT1 by DBC1. Nature.

[53] Abdelmawgoud H., El Awady R.R. (2017). Effect of Sirtuin 1 inhibition on matrix metalloproteinase 2 and Forkhead box O3a expression in breast cancer cells. Genes Dis..

[54] Sung J.-Y., Kim R., Kim J.-E., Lee J. (2010). Balance between SIRT1 and DBC1 expression is lost in breast cancer. Cancer Sci..

[55] Wu M., Wei W., Xiao X., Guo J., Xie X., Li L., Kong Y., Lv N., Jia W., Zhang Y. (2012). Expression of SIRT1 is associated with lymph node metastasis and poor prognosis in both operable triple-negative and non-triple-negative breast cancer. Med. Oncol..

[56] Jin X., Wei Y., Xu F., Zhao M., Dai K., Shen R., Yang S., Zhang N. (2018). SIRT1 promotes formation of breast cancer through modulating Akt activity. J. Cancer.

[57] Xu Y., Qin Q., Chen R., Wei C., Mo Q. (2018). SIRT1 promotes proliferation, migration, and invasion of breast cancer cell line MCF-7 by upregulating DNA polymerase delta1 (POLD1). Biochem. Biophys. Res. Commun..

[58] Ota H., Tokunaga E., Chang K., Hikasa M., Iijima K., Eto M., Kozaki K., Akishita M., Ouchi Y., Kaneki M. (2006). Sirt1 inhibitor, Sirtinol, induces senescence-like growth arrest with attenuated Ras-MAPK signaling in human cancer cells. Oncogene.

[59] Karbasforooshan H., Roohbakhsh A., Karimi G. (2018). SIRT1 and microRNAs: The role in breast, lung and prostate cancers. Exp. Cell Res..

[60] Zou Q., Tang Q., Pan Y., Wang X., Dong X., Liang Z., Huang D. (2017). MicroRNA-22 inhibits cell growth and metastasis in breast cancer via targeting of SIRT1. Exp. Ther. Med..

[61] Zhang X., Li Y., Wang D., Wei X. (2017). miR-22 suppresses tumorigenesis and improves radiosensitivity of breast cancer cells by targeting Sirt1. Biol. Res..

[62] Yamakuchi M., Ferlito M., Lowenstein C.J. (2008). miR-34a repression of SIRT1 regulates apoptosis. Proc. Natl. Acad. Sci. USA.

[63] Li L., Yuan L., Luo J., Gao J., Guo J., Xie X. (2013). MiR-34a inhibits proliferation and migration of breast cancer through down-regulation of Bcl-2 and SIRT1. Clin. Exp. Med..

[64] Ma W., Xiao G.G., Mao J., Lu Y., Song B., Wang L., Fan S., Fan P., Hou Z., Li J. (2015). Dysregulation of the miR-34a-SIRT1 axis inhibits breast cancer stemness. Oncotarget.

[65] Oberdoerffer P., Michan S., McVay M., Mostoslavsky R., Vann J., Park S.-K., Hartlerode A., Stegmuller J., Hafner A., Loerch P. (2008). SIRT1 redistribution on chromatin promotes genomic stability but alters gene expression during aging. Cell.

[66] Borra M.T., Smith B.C., Denu J.M. (2005). Mechanism of human SIRT1 activation by resveratrol. J. Biol. Chem..

[67] Wang R.-H., Sengupta K., Li C., Kim H.-S., Cao L., Xiao C., Kim S., Xu X., Zheng Y., Chilton B. (2008). Impaired DNA damage response, genome instability, and tumorigenesis in SIRT1 mutant mice. Cancer Cell.

[68] Zhang W., Luo J., Yang F., Wang Y., Yin Y., Strom A., Gustafsson J.Å., Guan X. (2016). BRCA1 inhibits AR-mediated proliferation of breast cancer cells through the activation of SIRT1. Sci. Rep..

[69] Yu Y., Liu Y., Zong C., Yu Q., Yang X., Liang L., Ye F., Nong L., Jia Y., Lu Y. (2016). Mesenchymal stem cells with Sirt1 overexpression suppress breast tumor growth via chemokine-dependent natural killer cells recruitment. Sci. Rep..

[70] Shi L., Tang X., Qian M., Liu Z., Meng F., Fu L., Wang Z., Zhu W.-G., Huang J.-D., Zhou Z. (2018). A SIRT1-centered circuitry regulates breast cancer stemness and metastasis. Oncogene.

[71] Li K., Ying M., Feng D., Du J., Chen S., Dan B., Wang C., Wang Y. (2016). Brachyury promotes tamoxifen resistance in breast cancer by targeting SIRT1. Biomed. Pharmacother..

[72] Choi H.-K., Cho K.B., Phuong N.T.T., Han C.Y., Han H.-K., Hien T.T., Choi H.S., Kang K.W. (2013). SIRT1-mediated FoxO1 deacetylation is essential for multidrug resistance-associated protein 2 expression in tamoxifen-resistant breast cancer cells. Mol. Pharm..

[73] Moore R.L., Faller D.V. (2013). SIRT1 represses estrogen-signaling, ligand-independent ERα-mediated transcription, and cell proliferation in estrogen-responsive breast cells. J. Endocrinol..

[74] Xu Z., Yang Y., Li B., Li Y., Xia K., Yang Y., Li X., Wang M., Li S., Wu H. (2018). Checkpoint suppressor 1 suppresses transcriptional activity of ERα and breast cancer cell proliferation via deacetylase SIRT1. Cell Death Dis..

[75] Lu R., Serrero G. (1999). Resveratrol, a natural product derived from grape, exhibits antiestrogenic activity and inhibits the growth of human breast cancer cells. J. Cell. Physiol..

[76] Elangovan S., Ramachandran S., Venkatesan N., Ananth S., Gnana-Prakasam J.P., Martin P.M., Browning D.D., Schoenlein P.V., Prasad P.D., Ganapathy V. (2011). SIRT1 is essential for oncogenic signaling by estrogen/estrogen receptor α in breast cancer. Cancer Res..

[77] Yao Y., Li H., Gu Y., Davidson N.E., Zhou Q. (2010). Inhibition of SIRT1 deacetylase suppresses estrogen receptor signaling. Carcinogenesis.

[78] Santolla M.F., Avino S., Pellegrino M., De Francesco E.M., De Marco P., Lappano R., Vivacqua A., Cirillo F., Rigiracciolo D.C., Scarpelli A. (2015). SIRT1 is involved in oncogenic signaling mediated by GPER in breast cancer. Cell Death Dis..

[79] Yi Y.W., Kang H.J., Kim H.J., Kong Y., Brown M.L., Bae I. (2013). Targeting Mutant p53 by a SIRT1 Activator YK-3-237 Inhibits the Proliferation of Triple-Negative Breast Cancer Cells. Oncotarget.

[80] Chung S.Y., Jung Y.Y., Park I.A., Kim H., Chung Y.R., Kim J.Y., Park S.Y., Im S.-A., Lee K.-H., Moon H.-G. (2016). Oncogenic role of SIRT1 associated with tumor invasion, lymph node metastasis, and poor disease-free survival in triple negative breast cancer. Clin. Exp. Metastasis.

[81] Urra F.A., Muñoz F., Córdova-Delgado M., Ramírez M.P., Peña-Ahumada B., Rios M., Cruz P., Ahumada-Castro U., Bustos G., Silva-Pavez E. (2018). FR58P1a; A new uncoupler of OXPHOS that inhibits migration in triple-negative breast cancer cells via Sirt1/AMPK/β1-integrin pathway. Sci. Rep..

[82] Thiery J.P., Acloque H., Huang R.Y.J., Nieto M.A. (2009). Epithelial-mesenchymal transitions in development and disease. Cell.

[83] Simic P., Williams E.O., Bell E.L., Gong J.J., Bonkowski M., Guarente L. (2013). SIRT1 suppresses the epithelial-to-mesenchymal transition in cancer metastasis and organ fibrosis. Cell Rep..

[84] Eades G., Yao Y., Yang M., Zhang Y., Chumsri S., Zhou Q. (2011). miR-200a regulates SIRT1 expression and epithelial to mesenchymal transition (EMT)-like transformation in mammary epithelial cells. J. Biol. Chem..

[85] Jin M.-S., Hyun C.L., Park I.A., Kim J.Y., Chung Y.R., Im S.-A., Lee K.-H., Moon H.-G., Ryu H.S. (2016). SIRT1 induces tumor invasion by targeting epithelial mesenchymal transition-related pathway and is a prognostic marker in triple negative breast cancer. Tumor Biol..

[86] Mahajan S.S., Leko V., Simon J.A., Bedalov A. (2011). Sirtuin modulators. Handb. Exp. Pharmacol..

[87] Villalba J.M., Alcaín F.J. (2012). Sirtuin activators and inhibitors. Biofactors.

[88] Mellini P., Valente S., Mai A. (2015). Sirtuin modulators: An updated patent review (2012–2014). Expert Opin. Ther. Pat..

[89] Bai X., Yao L., Ma X., Xu X. (2018). Small Molecules as SIRT Modulators. Mini Rev. Med. Chem..

[90] Milne J.C., Denu J.M. (2008). The Sirtuin family: Therapeutic targets to treat diseases of aging. Curr. Opin. Chem. Biol..

[91] Kozako T., Suzuki T., Yoshimitsu M., Arima N., Honda S., Soeda S. (2014). Anticancer agents targeted to sirtuins. Molecules.

[92] Hu J., Jing H., Lin H. (2014). Sirtuin inhibitors as anticancer agents. Future Med. Chem..

[93] Timmers S., Auwerx J., Schrauwen P. (2012). The journey of resveratrol from yeast to human. Aging.

[94] Milne J.C., Lambert P.D., Schenk S., Carney D.P., Smith J.J., Gagne D.J., Jin L., Boss O., Perni R.B., Vu C.B. (2007). Small molecule activators of SIRT1 as therapeutics for the treatment of type 2 diabetes. Nature.

[95] Yoon Y.K., Ali M.A., Wei A.C., Choon T.S., Osman H., Parang K., Shirazi A.N. (2014). Synthesis and evaluation of novel benzimidazole derivatives as sirtuin inhibitors with antitumor activities. Bioorg. Med. Chem..

[96] Mellini P., Kokkola T., Suuronen T., Salo H.S., Tolvanen L., Mai A., Lahtela-Kakkonen M., Jarho E.M. (2013). Screen of pseudopeptidic inhibitors of human sirtuins 1–3: Two lead compounds with antiproliferative effects in cancer cells. J. Med. Chem..

[97] Alvala M., Bhatnagar S., Ravi A., Jeankumar V.U., Manjashetty T.H., Yogeeswari P., Sriram D. (2012). Novel acridinedione derivatives: Design, synthesis, SIRT1 enzyme and tumor cell growth inhibition studies. Bioorg. Med. Chem. Lett..

[98] Wang J., Kim T.H., Ahn M.Y., Lee J., Jung J.H., Choi W.S., Lee B.M., Yoon K.S., Yoon S., Kim H.S. (2012). Sirtinol, a class III HDAC inhibitor, induces apoptotic and autophagic cell death in MCF-7 human breast cancer cells. Int. J. Oncol..

[99] Rotili D., Tarantino D., Nebbioso A., Paolini C., Huidobro C., Lara E., Mellini P., Lenoci A., Pezzi R., Botta G. (2012). Discovery of salermide-related sirtuin inhibitors: Binding mode studies and antiproliferative effects in cancer cells including cancer stem cells. J. Med. Chem..

[100] Peck B., Chen C.-Y., Ho K.-K., Di Fruscia P., Myatt S.S., Coombes R.C., Fuchter M.J., Hsiao C.-D., Lam E.W.-F. (2010). SIRT inhibitors induce cell death and p53 acetylation through targeting both SIRT1 and SIRT2. Mol. Cancer Ther..

